# Evaluating Patient Perspectives of Provider Professionalism on Twitter in an Academic Obstetrics and Gynecology Clinic: Patient Survey

**DOI:** 10.2196/jmir.8056

**Published:** 2018-03-12

**Authors:** Rosalyn E Maben-Feaster, R Brent Stansfield, AnneMarie Opipari, Maya M Hammoud

**Affiliations:** ^1^ Department of Obstetrics and Gynecology University of Michigan Ann Arbor, MI United States; ^2^ Graduate Medical Education Wayne State University School of Medicine Detroit, MI United States; ^3^ University of Michigan Medical School Ann Arbor, MI United States

**Keywords:** patients, social networking sites, professionalism, surveys and questionnaires, perception, Twitter

## Abstract

**Background:**

One-third of Americans use social media websites as a source of health care information. Twitter, a microblogging site that allows users to place 280-character posts—or tweets—on the Web, is emerging as an important social media platform for health care. However, most guidelines on medical professionalism on social media are based on expert opinion.

**Objective:**

This study sought to examine if provider Twitter profiles with educational tweets were viewed as more professional than profiles with personal tweets or a mixture of the two, and to determine the impact of provider gender on perceptions of professionalism in an academic obstetrics and gynecology clinic.

**Methods:**

This study randomized obstetrics and gynecology patients at the University of Michigan Von Voigtlander Clinic to view one of six medical provider Twitter profiles, which differed in provider gender and the nature of tweets. Each participant answered 10 questions about their perception of the provider’s professionalism based on the Twitter profile content.

**Results:**

The provider profiles with educational tweets alone received higher mean professionalism scores than profiles with personal tweets. Specifically, the female and male provider profiles with exclusively educational tweets had the highest and second highest overall mean professionalism ratings at 4.24 and 3.85, respectively. In addition, the female provider profiles received higher mean professionalism ratings than male provider profiles with the same content. The female profile with mixed content received a mean professionalism rating of 3.38 compared to 3.24 for the male mixed-content profile, and the female profile with only personal content received a mean professionalism rating of 3.68 compared to 2.68 for the exclusively personal male provider profile.

**Conclusions:**

This study showed that in our obstetrics and gynecology clinic, patients perceived providers with educational profiles as more professional than those with a mixture of educational and personal tweets or only personal tweets. It also showed that our patient population perceived the female provider with educational tweets to be the most professional. This study will help inform the development of evidence-based guidelines for social media use in medicine as it adds to the growing body of literature examining professionalism and social media.

## Introduction

Social media is a form of online communication, such as websites for social networking and microblogging, through which users can create online communities to share information, ideas, personal messages, and other content [1,2]. In a 2014 study, the Pew Research Center showed that 74% of Americans on the Internet use social media sites [3]. Furthermore, one-third of Americans use social media for health care discussions, according to a study by PricewaterhouseCoopers [4]. Facebook, a platform for users to share their stories and connect to other people, is the most popular site, with 1.04 billion daily users worldwide [5,6]. Other commonly used tools include YouTube, which is a social media platform that allows users to discover, watch, and share videos, and Twitter, a microblogging site that allows users to place 280-character posts—or tweets—on the Web [7,8]. YouTube has approximately one billion users worldwide, while Twitter has 320 million active users [7,8]. A Health Research Institute consumer survey showed that Facebook and YouTube are the most commonly used social media tools for consumers to view health-related information [4]. However, Twitter is emerging as one of the leading social media platforms for health care. It has shown significant growth, with 460,000 new accounts created on average per day [4]. Social media offers a number of opportunities for health care organizations and health care professionals. Twitter alone has been shown to have more than 140 different uses in health care [9] including disaster alerting and response, diabetes management, and drug safety [10]. On an individual level, 90% of physicians use social media for personal reasons and 67% use it professionally [11,12]. Thus, it serves as a way for health care providers to provide health education, connect with patients, and increase market share via a unique platform that allows information to be disseminated beyond the capabilities of traditional digital media—such as webpages—which makes it an attractive tool for organizations and individual professionals [13]. However, this capacity to reach a large audience also increases the likelihood of unknown users interacting with the site [13].

Many professional organizations such as the American Medical Association, the Federation for State Medical Boards, the American Board of Internal Medicine, the American College of Obstetricians and Gynecologists, and many individual health care organizations have developed guidelines to help health care organizations and providers create social media presences that discourage posting of inaccurate information, avoid damaging professional identities, preserve patient privacy and the provider-patient relationship, and avoid ligation [9]. However, the bulk of these guidelines are created based on expert opinion. Thus, there is a need for research into the evolution of professionalism in the digital era—also known as e-professionalism [14]. There are numerous papers discussing the issue of professionalism in social media. However, there is limited data on how a provider’s social media presence impacts a patient’s perception of that provider’s professionalism. A 2014 study by Jain and colleagues at the University of Michigan evaluated what medical students, doctors, and the public felt was unprofessional for medical students to post on Facebook [15]. The results showed that the public and faculty had lower thresholds for what was considered appropriate and that this was also related to how comfortable they would be with these students caring for them [15]. Interestingly, doctors, females, and older individuals were less permissive regarding the appropriateness of content [15]. Clyde and colleagues examined how a physician’s Facebook profile can impact a potential patient’s impression of that provider’s professionalism [16] and found that personal profiles containing healthy behavior were rated as most professional, followed by profiles with strictly professional content. Unhealthy personal profiles were rated as least professional [16]. In addition, profiles of female providers were rated more professional across all profile types [16].

However, data are lacking on the public’s perception of provider professionalism in the context of Twitter. There is also limited research on how obstetrics and gynecology patients and providers use social media and how that use relates to medical professionalism. Our study sought to help close this gap and inform the discussion on how health care providers should use social media. Specifically, we aimed to determine whether a provider Twitter profile with educational tweets was viewed as more professional than a Twitter profile with personal tweets or a mixture of the two, as determined by patients in an academic obstetrics and gynecology clinic. It also sought to determine whether these patients would perceive the Twitter profiles of female providers to be more professional than the Twitter profiles of male providers, regardless of the content of the profile.

## Methods

### Ethics

This study, including the introduction letter and survey instrument, was reviewed and determined exempt by the Medical School Institutional Review Board at the University of Michigan. A signed consent was not required, but each survey began with a letter explaining that this was a voluntary research study. This letter also explained how the participant’s confidentiality would be maintained, that participation would not affect their care, and that the participant could receive the study results at study completion if they wished. Finally, the letter also offered them the opportunity to be entered in a draw for one of five gift certificates valued at US $100. The contact information for those participants that entered the draw was kept separate from the survey to protect their confidentiality. Participants were able to withdraw by their own request at any time by simply not submitting the survey.

### Eligibility Criteria and Study Setting

In July 2012, 200 surveys were distributed to women receiving care in the Obstetrics and Gynecology clinic at the University of Michigan Von Voigtlander Women’s Hospital in Ann Arbor, Michigan. In order to participate, patients had to be at least 18 years or older and able to read English.

### Study Design

Patients were randomized to receive one of six different printed survey packets at the time of appointment check-in. Each survey packet included a letter describing the study, questions regarding demographics and social media use, a color screenshot of one of six different medical provider Twitter profiles, and a 10-question survey instrument looking at the patient’s attitude about the professionalism of the provider whose profile was viewed. Participants submitted the completed packet prior to leaving the clinic.

### Medical Provider Twitter Profile Creation

Each of the six profiles was created on Twitter. The profiles all had the same profile picture (stethoscope clipart), background, and contained 8 tweets. However, the profiles differed with regard to profile name and content. Specifically, the names were selected to reflect the profiles of three female physicians and 3 male physicians. Twitter does not allow the creation of multiple profiles with the exact same name, thus the female provider profiles had three permutations of a similar first name but the same last name (Ashley, Ashlee, and Ashleigh Scott, MD). The male profiles followed a similar pattern (Jahn, John, and Jon Scott, MD). For each gender, the Twitter profiles were designed to fit into one of three categories: educational only (Ashlee and John), personal only (Ashley and Jahn), or a 50/50 mixture of the two (Ashleigh and Jon). The tweets used were adapted from tweets posted by self-identified obstetrician gynecologists on Twitter. The 8 tweets selected were the same within the content group regardless of gender. For example, the male provider’s educational only Twitter profile had the exact same tweets as the female provider’s educational only Twitter profile (see Multimedia Appendix 1).

### Rating Scale for Provider Professionalism

Patients were asked to rate 10 statements about each provider’s professionalism based on their tweets. The professionalism statements were developed from the American Board of Internal Medicine’s 10 professional responsibilities: professional competence, honesty with patients, patient confidentiality, maintaining appropriate relationships with patients, improving quality of care, improving access to care, just distribution of finite resources, scientific knowledge, maintaining trust by managing conflicts of interest, and professional responsibilities [17,18].

A 7-point scale was used to rate the responses to each of the 10 professionalism statements:

1. This provider seems like he/she has the skills to take care of my health care needs (skill).
2. This provider seems like he/she would be honest with me (honesty).
3. This provider seems like he/she would keep my health information private (privacy).
4. This provider seems like he/she would maintain appropriate boundaries with patients and other health care providers (boundary).
5. This provider seems like he/she would work to improve the quality of health care (work to improve).
6. This provider seems like he/she would work to provide good access to health care (access).
7. This provider seems like he/she would use health resources fairly and appropriately (resource use).
8. This provider seems like he/she knows what he/she is doing (competent).
9. This provider seems like he/she knows how to maintain the patient’s trust while balancing conflicting interests (trust).
10. This provider seems like he/she follows through on his/her professional responsibilities (responsible).

### Statistical Analysis

In the Johnson study, there was a nonparametric correlation between perceived credibility and whether a teacher posted social or scholarly content [19]. Given the use of nonparametric statistics in the Johnson study, the effect size for a power analysis was difficult to estimate, but we conservatively estimated a moderate effect according to Cohen (*d*=0.3). We calculated a power of 80% with 87 participants [19]. Adding gender and its interaction, a sample size of 100 gave a power of 80% to detect an overall effect size of eta squared=0.105 [19]. We predicted that if there was an effect of professionalism and gender in an interaction, and these together explained 10.5% of the variance, we would find the effect 80 times out of 100 experiments [19]. The data was analyzed using analysis of variance, with a *P* value of .05 considered to be statistically significant. The data from all participants who submitted answers to the professionalism section of the survey were included in the analysis.

## Results

During the 4 weeks of survey collection, 200 surveys were distributed and 134 were returned completed, giving a response rate of 67%. We were unable to collect data about the patients who opted not to complete the survey given how the packets were distributed.

The demographics of the population surveyed reflected our clinic’s overall population. In general, our patients are primarily young, college-educated, married, non-Hispanic white women (Table 1). The majority (127/134, 94.8%) of participants were under age 50, three-quarters (99/134, 73.9%) identified as white, two-thirds (87/134, 64.9%) were married, and the majority (120/134, 89.6%) completed at least some college (Table 1). With regard to annual household income, 34.3% (46/134) made more than US $75,000 per year and 34.3% (46/134) made less than US $50,000 per year.

With respect to social media use, 91.0% (122/134) of participants used social media, with Facebook, blogs, and Twitter being the most popular. However, only 20.9% (28/134) of participants used social media for health care purposes. The uses indicated by our participants included gaining knowledge about conditions or treatments, support groups, sharing experiences, and receiving or giving advice.

When examining the ratings of the 10 professionalism statements (ie, skill, honesty, privacy, boundary, work to improve, access, resource use, competent, trust, responsible), we found a statistically significant difference for 6 of the 10 dimensions, including skill, work to improve, access, resource use, competent, and responsible (Table 2).

**Table 1:** Demographics of study participants (N=134).

Characteristics		n (%)
**Race/Ethnicity**		
	White	99 (74)
	Black	11 (8)
	Asian	13 (10)
	American Indian or Alaskan Native	2 (1)
	Other	5 (4)
	Hispanic or Latino	5 (4)
**Marital status**		
	Married	87 (65)
	Single	39 (29)
	Separated	2 (1)
	Divorced	2 (1)
	Widowed	1 (1)
**Age**		
	18-29	52 (39)
	30-49	75 (56)
	50-64	9 (7)
	≥65	3 (2)
**Education level**		
	Not a high school graduate	4 (3)
	High school graduate	10 (7)
	Some college	120 (90)

**Table 2:** Survey results: mean ratings for professionalism statements by profile types. Ratings based on 7-point scale.

	Female, education (Ashlee)	Male, education (John)	Female, personal (Ashley)	Male, personal (Jahn)	Female, mixed (Ashleigh)	Male, mixed (Jon)
Skill	4.14	3.65	3.30	2.26	3.18	2.58
Honesty	4.05	3.47	3.96	2.91	3.71	3.53
Privacy	3.90	3.29	3.65	2.78	3.24	3.42
Boundary	4.10	3.65	3.91	2.65	3.06	3.26
Work to improve	4.40	4.59	3.57	2.50	3.65	3.16
Access	4.50	4.59	3.78	2.78	3.76	3.21
Resource use	4.45	3.94	3.78	2.74	3.88	3.28
Competent	4.29	3.82	3.65	2.48	3.29	3.82
Trust	4.15	3.59	3.57	2.78	3.12	3.00
Responsible	4.45	3.88	3.61	2.87	2.94	3.11

**Figure 1:**
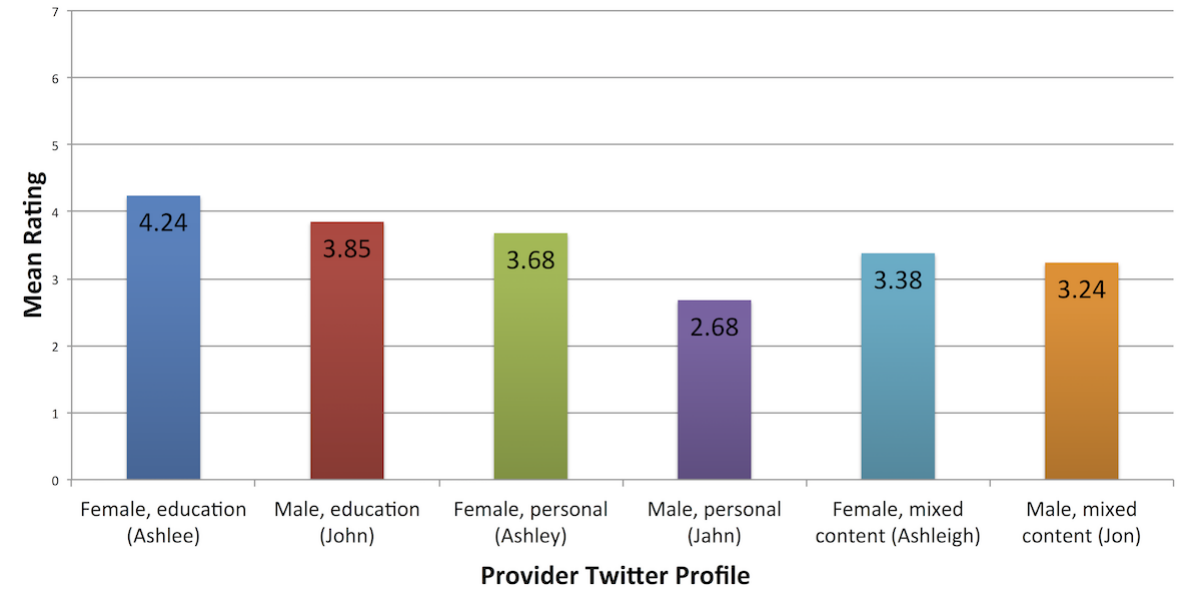
Survey results: overall mean ratings by profile type.

In general, profiles with educational tweets received higher professionalism ratings compared to those with personal tweets (Table 2). Also, female providers received higher ratings than male providers with similar profiles (Table 2). Specifically, the study population rated the female provider with educational tweets (Ashlee Scott, MD) as more professional than the male provider with personal tweets (Jahn Scott, MD) and the male provider with a mixture of educational and personal tweets (Jon Scott, MD) in each of these dimensions (Table 2). The female provider with educational tweets was also rated more responsible than the female provider with a mixture of educational and personal tweets (Ashleigh Scott, MD) (Table 2). Rating items were highly intercorrelated, suggesting that the ratings reflect the same construct or a set of highly related constructs. This intercorrelation justified computing a mean rating across items as an estimate of the participant’s perception of the physician's overall quality. At 5% significance level, the data provide sufficient evidence to conclude that a difference exists between the mean rating among the six profiles (*P*=.002). In addition, at a 5% significance level, the data provide sufficient evidence to conclude that mean ratings for the female educational Twitter profile were higher than those for the male personal profile (*P*=.001) and the male mixed profile (*P*=.03) (Figure 1). Specifically, the female provider profile with educational tweets had a mean rating of 4.243 compared to 3.847 for the male provider profile with the same tweets (Figure 1). The mean rating for the female provider profile with personal tweets was 3.678 compared to 2.675 for the male provider profile with the same tweets (Figure 1). Finally, the female provider profile with a mixture of educational and personal tweets had a mean rating of 3.383 compared to a mean rating of 3.237 for the male provider profile with the same tweets (Figure 1).

## Discussion

### Principal Findings

Many physicians use social media for both personal and business uses [11,12]. However, data are limited on how a provider’s social media profile impacts the patient’s perception of that provider’s professionalism. Our study sought to help close this gap in the obstetrics and gynecology patient population. In this study, obstetrics and gynecology patients were randomized to view a screenshot from the Twitter profile of one of six different fictitious providers and then rate their professionalism based on the content of the tweets viewed. In general, profiles with educational tweets received higher professionalism ratings from our study participants than profiles with mixed content or purely personal tweets. Specifically, the female and male provider profiles with exclusively educational tweets had the highest and second highest overall mean professionalism ratings at 4.24 and 3.85 respectively. This is consistent with what would have been expected based on the traditional definition of medical professionalism outside the context of social media. In keeping with this theory, the mean professionalism score among the male provider profiles decreased as the content of the profile became more personal in nature with the exclusively personal Twitter profile having the lowest professionalism score of 2.68. However, among the female provider profiles, a slightly different pattern was seen. The purely educational Twitter profile had the highest professionalism rating. However, the female provider profile with the second highest mean professionalism rating was actually the provider profile with exclusively personal tweets at 3.68 rather than the mixed content Twitter profile at 3.38. This discrepancy may be driven by the fact that the patient population surveyed was all female and these patients may have identified more with the persona of the female provider with personal tweets, resulting in a higher professionalism score. This may also explain why the female provider profiles had higher mean professionalism scores when compared to the male provider profiles with the same content.

### Limitations

There were a number of limitations to this study. First, the study was conducted in an obstetrics and gynecology clinic and all of the study participants identified as female. This may have biased our finding that female providers were viewed as more professional since the patients may have identified more with providers of the same gender. Interestingly, Jain and colleagues studied medical student Facebook profiles and found that female participants tended to be less permissive regarding the appropriateness of profile content [15]. The Facebook-based professionalism study conducted by Clyde and colleagues also found that those surveyed viewed female providers as more professional and their study population included both female and male participants [16]. This suggests that more research is needed to determine why female gender alone creates a greater perception of professionalism. The majority of the participants in this study were under age 50 (95%), which limits the generalizability of these findings to an older population. However, Pew Research Center data suggest that Twitter use is less common in persons over age 50 [3]. Their data show that only 11% of Internet users over age 50 use Twitter, compared to 30% of Internet users under age 50 [3]. This suggests that in older populations, Twitter is less likely to be used for health-related information and provider selection. Finally, at the time of study design and implementation, there was no pre-existing measurement scale available for evaluation of perceptions of professionalism. Therefore, we developed our own survey instrument, which had not been validated in other studies. However, the ratings were highly intercorrelated, suggesting that the ratings reflect the same or a highly similar construct, which supports the instrument’s validity. Since conducting our study, another scale to measure perceived professionalism was developed—the First Impressions of Medical Professionalism (FIMP) scale [16]. This creates an opportunity to confirm the findings of our study using a different instrument.

### Conclusion

When patients view a provider’s profile on a social media platform, they do not necessarily discern between whether the provider’s profile is for personal or business use. In addition, it is difficult to control who may be able to view a given profile. Professionalism serves as the foundation of the patient-provider relationship. If it is eroded prior to the patient entering the clinic, due to a provider’s social media presence, this can have implications with regard to care. However, social media platforms also allow medical providers to interact and reach patients in a unique way, which may improve care. Given this delicate balance, it is important to expand the body of knowledge on medical professionalism in the context of social media. In an academic obstetrics and gynecology clinic, we found patients identifying as female perceived providers with purely educational Twitter feeds as more professional than those with mixed content or purely personal tweets. To our knowledge, this is the first study to look at the issue of patient-perceived professionalism among providers who tweet and the impact of provider gender on this perception. It provides a foundation for further research into how this technology impacts our ability to educate patients and each other.

## Appendix

Multimedia Appendix 1 Medical provider Twitter profile screenshots as viewed by study participants.
