# Supplementary material for: Evaluating Patient Perspectives of Provider Professionalism on Twitter in an Academic Obstetrics and Gynecology Clinic: Patient Survey
Source: J Med Internet Res. 2018 Mar 12;20(3):e78. doi: 10.2196/jmir.8056 (PMC5869178; doi:10.2196/jmir.8056)
Supplement: Multimedia Appendix 1 [file jmir_v20i3e78_app1.pdf]

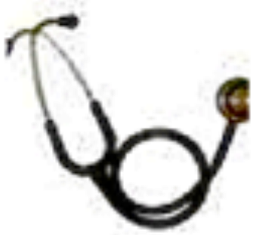

Ashley Scott, MD

@AshleyScottMD

Edit your profile

8 TWEETS

3 FOLLOWING

0 FOLLOWERS

- **Tweets** >
- Following >
- Followers >
- Favorites >
- Requests >
- Lists >

twitter
© 2012 Twitter About Help Terms Privacy
Blog Status Apps Resources Jobs
Advertisers Businesses Media Developers

Tweets

Ashley Scott, MD @AshleyScottMD — 31 Jan
Excited for tonight! Happy Bday!
Expand

Ashley Scott, MD @AshleyScottMD — 22 Jan
Celebrating hubby's birthday and hosting son's counselor colleagues from camp
Expand

Ashley Scott, MD @AshleyScottMD — 22 Jan
Preparing for the youngest to graduate from high school - wow, how time flies...
Expand

Ashley Scott, MD @AshleyScottMD — 11 Jan
Get a travel guitar. Fits in overhead locker. Really cool! Take mine everywhere.
Expand

Ashley Scott, MD @AshleyScottMD — 10 Jan
Fact about Dr. S: When not taking care of her patients, she enjoys getting outside.
Expand

Ashley Scott, MD @AshleyScottMD — 8 Jan
In San Francisco, grabbing a bite to eat at Tony's Pizza
Expand

Ashley Scott, MD @AshleyScottMD — 3 Jan
Eating donuts and drinking coffee now
Expand

Ashley Scott, MD @AshleyScottMD — 30 Dec
Was reminded that with the upcoming holiday that she has SO MUCH for which to be grateful. :D

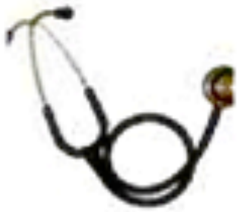

Ashleigh Scott, MD

@AshleighScottMD

Edit your profile

8 TWEETS

0 FOLLOWING

0 FOLLOWERS

- Tweets
- Following
- Followers
- Favorites
- Requests
- Lists

twitter

© 2012 Twitter About Help Terms Privacy
Blog Status Apps Resources Jobs
Advertisers Businesses Media Developers

Tweets

Ashleigh Scott, MD @AshleighScottMD 31 Jan
Excited for tonight! Happy Bday!
Expand

Ashleigh Scott, MD @AshleighScottMD 22 Jan
Celebrating hubby's birthday and hosting son's counselor colleagues from camp
Expand

Ashleigh Scott, MD @AshleighScottMD 22 Jan
Exercise my improve cognitive skills in older population: medscape.com/viewarticle/71...
Expand

Ashleigh Scott, MD @AshleighScottMD 11 Jan
Get a travel guitar. Fits in overhead locker. Really cool! Take mine everywhere.
Expand

Ashleigh Scott, MD @AshleighScottMD 10 Jan
Midlife Weight Loss Cuts Heart Risk: nyti.ms/lrzx7VJ
Expand

Ashleigh Scott, MD @AshleighScottMD 8 Jan
Weight Guidelines May Be High for Severely Obese Moms-to-Be - Yahoo! News yhoo.it/h2fLXV
Expand

Ashleigh Scott, MD @AshleighScottMD 3 Jan
Eating donuts and drinking coffee now
Expand

Ashleigh Scott, MD @AshleighScottMD 30 Dec
Flu Vaccine Protects Pregnant Woman's Baby And Does Not Cause Miscarriage mnt.to/42vP via @mnt_flu7
[illegible]

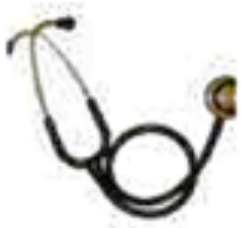

Ashlee Scott, MD

@AshleeScottMD

Edit your profile

8 TWEETS

0 FOLLOWING

0 FOLLOWERS

Tweets

Following

Followers

Favorites

Requests

Lists

twitter

© 2012 Twitter About Help Terms Privacy Blog Status Apps Resources Jobs Advertisers Businesses Media Developers

Tweets

Ashlee Scott, MD @AshleeScottMD 31 Jan
Exercising During Pregnancy Protects Offspring Against Long-Term Neurodegenerative Diseases mnt.to/3ZVf via @mnt_pregnancy
Expand

Ashlee Scott, MD @AshleeScottMD 22 Jan
Simple ovarian cysts not linked to cancer in postmenopausal women: bit.ly/bfeyzh
Expand

Ashlee Scott, MD @AshleeScottMD 22 Jan
Exercise may improve cognitive skills in older population: medscape.com/viewarticle/71...
Expand

Ashlee Scott, MD @AshleeScottMD 11 Jan
Study Suggest Link Between Diet Sodas, Preterm Delivery - Yahoo! News yhoo.it/9iO3kf
Expand

Ashlee Scott, MD @AshleeScottMD 10 Jan
Midlife Weight Loss Cuts Heart Risk: nyti.ms/rzx7VJ
Expand

Ashlee Scott, MD @AshleeScottMD 8 Jan
Weight Guidelines May Be High for Severely Obese Moms-to-Be - Yahoo! News yhoo.it/h2fLXV
Expand

Ashlee Scott, MD @AshleeScottMD 3 Jan
BPA Exposure In Womb Linked to Behavior Problems in Young Girls mnt.to/42w5 via @mnt_pregnancy
Expand

Ashlee Scott, MD @AshleeScottMD 30 Dec
Flu Vaccine Protects Pregnant Woman's Baby And Does Not Cause Miscarriage mnt.to/42vP via @mnt_flu

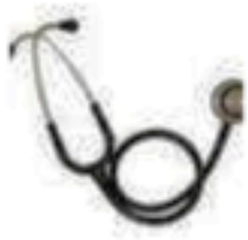

John Scott, MD

@JohnScottMD

Edit your profile

8 TWEETS

0 FOLLOWING

0 FOLLOWERS

Tweets

Following

Followers

Favorites

Requests

Lists

twitter

© 2012 Twitter About Help Terms Privacy
Blog Status Apps Resources Jobs
Advertisers Businesses Media Developers

Tweets

John Scott, MD @JohnScottMD — 31 Jan
Exercising During Pregnancy Protects Offspring Against Long-Term Neurodegenerative Diseases mnt.to/3ZVf via @mnt_pregnancy
Expand

John Scott, MD @JohnScottMD — 22 Jan
Simple ovarian cysts not linked to cancer in postmenopausal women: bit.ly/bfeyzh
Expand

John Scott, MD @JohnScottMD — 22 Jan
Exercise may improve cognitive skills in older population: medscape.com/viewarticle/71...
Expand

John Scott, MD @JohnScottMD — 11 Jan
Study Suggest Link Between Diet Sodas, Preterm Delivery - Yahoo! News yhoo.it/9iO3kf
Expand

John Scott, MD @JohnScottMD — 10 Jan
Midlife Weight Loss Cuts Heart Risk: nyti.ms/rzx7VJ
Expand

John Scott, MD @JohnScottMD — 8 Jan
Weight Guidelines May Be High for Severely Obese Moms-to-Be - Yahoo! News yhoo.it/h2fLXV
Expand

John Scott, MD @JohnScottMD — 3 Jan
BPA Exposure In Womb Linked to Behavior Problems In Young Girls mnt.to/42w5 via @mnt_pregnancy
Expand

John Scott, MD @JohnScottMD — 30 Dec
Flu Vaccine Protects Pregnant Woman's Baby And Does Not Cause Miscarriage mnt.to/42vP via @mnt_flu7

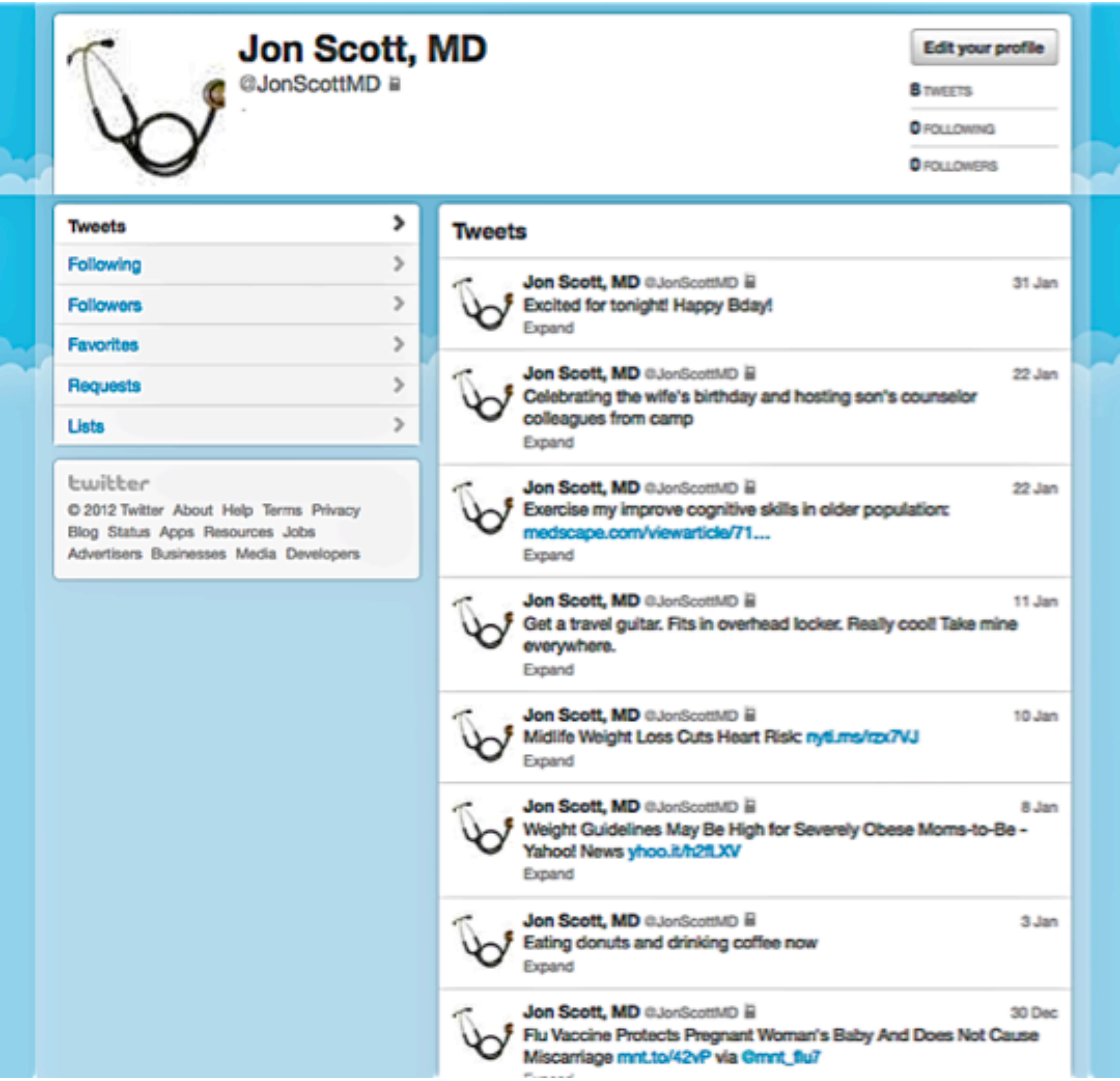
Jon Scott, MD
@JonScottMD
Edit your profile
8 TWEETS
0 FOLLOWING
0 FOLLOWERS
Tweets
Following
Followers
Favorites
Requests
Lists
twitter
© 2012 Twitter About Help Terms Privacy
Blog Status Apps Resources Jobs
Advertisers Businesses Media Developers
Tweets
Jon Scott, MD @JonScottMD 31 Jan
Excited for tonight! Happy Bday!
Expand
Jon Scott, MD @JonScottMD 22 Jan
Celebrating the wife's birthday and hosting son's counselor colleagues from camp
Expand
Jon Scott, MD @JonScottMD 22 Jan
Exercise my improve cognitive skills in older population: medscape.com/viewarticle/71....
Expand
Jon Scott, MD @JonScottMD 11 Jan
Get a travel guitar. Fits in overhead locker. Really cool! Take mine everywhere.
Expand
Jon Scott, MD @JonScottMD 10 Jan
Midlife Weight Loss Cuts Heart Risk: nyti.ms/rzx7VJ
Expand
Jon Scott, MD @JonScottMD 8 Jan
Weight Guidelines May Be High for Severely Obese Moms-to-Be - Yahoo! News yhoo.it/h2fLXV
Expand
Jon Scott, MD @JonScottMD 3 Jan
Eating donuts and drinking coffee now
Expand
Jon Scott, MD @JonScottMD 30 Dec
Flu Vaccine Protects Pregnant Woman's Baby And Does Not Cause Miscarriage mnt.to/42vP via @mnt_flu7

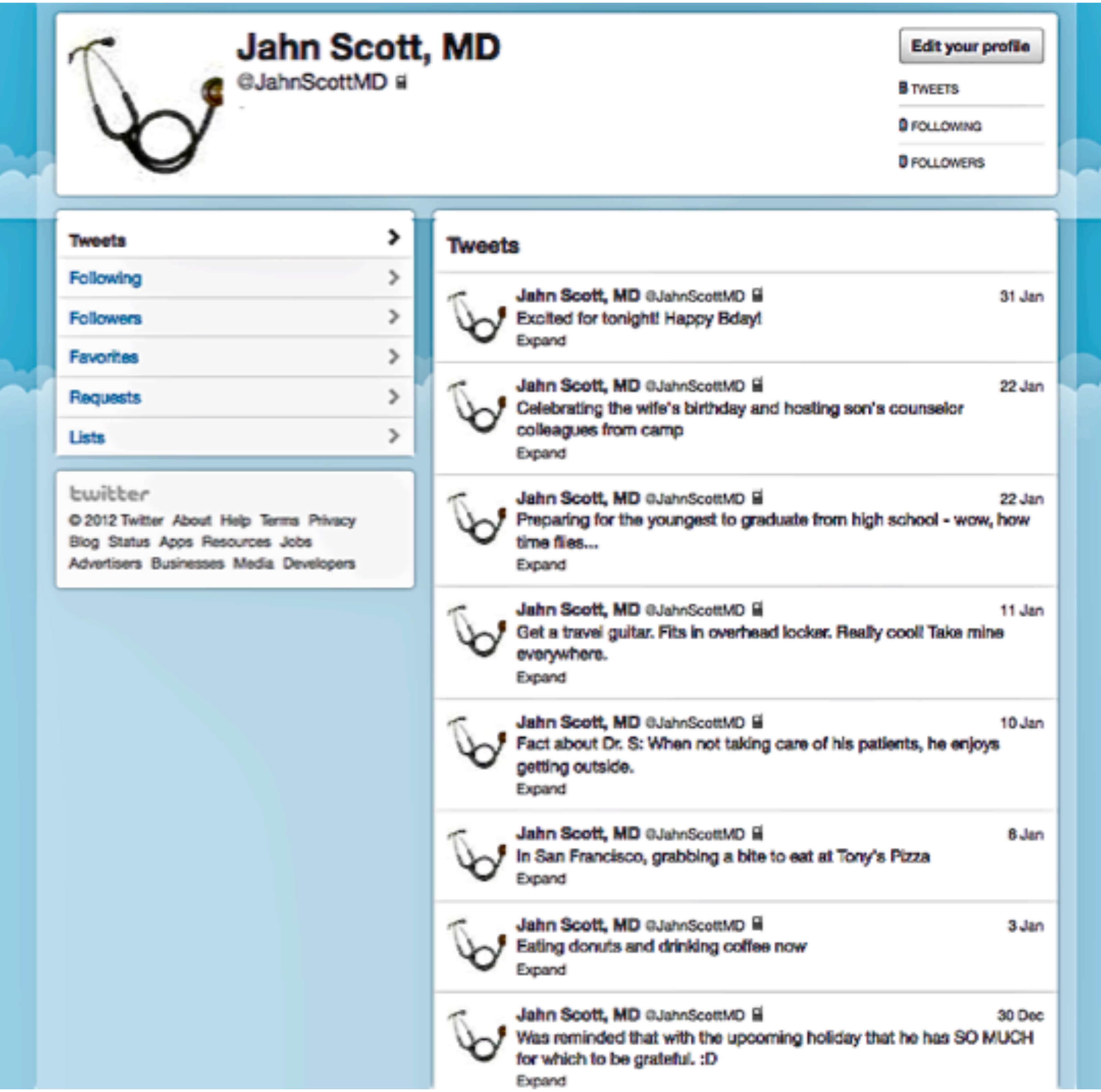
Jahn Scott, MD
@JahnScottMD
Edit your profile
8 TWEETS
0 FOLLOWING
0 FOLLOWERS
Tweets
Following
Followers
Favorites
Requests
Lists
twitter
© 2012 Twitter About Help Terms Privacy
Blog Status Apps Resources Jobs
Advertisers Businesses Media Developers
Tweets
Jahn Scott, MD @JahnScottMD 31 Jan
Excited for tonight! Happy Bday!
Expand
Jahn Scott, MD @JahnScottMD 22 Jan
Celebrating the wife's birthday and hosting son's counselor colleagues from camp
Expand
Jahn Scott, MD @JahnScottMD 22 Jan
Preparing for the youngest to graduate from high school - wow, how time flies...
Expand
Jahn Scott, MD @JahnScottMD 11 Jan
Get a travel guitar. Fits in overhead locker. Really cool! Take mine everywhere.
Expand
Jahn Scott, MD @JahnScottMD 10 Jan
Fact about Dr. S: When not taking care of his patients, he enjoys getting outside.
Expand
Jahn Scott, MD @JahnScottMD 8 Jan
In San Francisco, grabbing a bite to eat at Tony's Pizza
Expand
Jahn Scott, MD @JahnScottMD 3 Jan
Eating donuts and drinking coffee now
Expand
Jahn Scott, MD @JahnScottMD 30 Dec
Was reminded that with the upcoming holiday that he has SO MUCH for which to be grateful. :D
Expand
